# *In Vitro* characterization of the endocrine disrupting effects of per- and poly-fluoroalkyl substances (PFASs) on the human androgen receptor

**DOI:** 10.1016/j.jhazmat.2022.128243

**Published:** 2022-01-10

**Authors:** Phum Tachachartvanich, Ettayapuram Ramaprasad Azhagiya Singam, Kathleen A. Durkin, J. David Furlow, Martyn T. Smith, Michele A. La Merrill

**Affiliations:** a Department of Environmental Toxicology, University of California, Davis 95616, CA, USA; b Laboratory of Environmental Toxicology, Chulabhorn Research Institute, Bangkok 10210, Thailand; c Molecular Graphics and Computation Facility, College of Chemistry, University of California, Berkeley 94720, CA, USA; d Department of Neurobiology, Physiology and Behavior, University of California, Davis 95616, CA, USA; e Division of Environmental Health Sciences, School of Public Health, University of California, Berkeley 94720, CA, USA

**Keywords:** PFASs, Endocrine disruptors, Antiandrogens, Human androgen receptor

## Abstract

Per- and poly-fluoroalkyl substances (PFASs) are used extensively in a broad range of industrial applications and consumer products. While a few legacy PFASs have been voluntarily phased out, over 5000 PFASs have been produced as replacements for their predecessors. The potential endocrine disrupting hazards of most emerging PFASs have not been comprehensively investigated. *In silico* molecular docking to the human androgen receptor (hAR) combined with machine learning techniques were previously applied to 5206 PFASs and predicted 23 PFASs bind the hAR. Herein, the *in silico* results were validated *in vitro* for the five candidate AR ligands that were commercially available. Three manufactured PFASs namely (9-(nonafluorobutyl)– 2,3,6,7-tetrahydro-1 H,5 H,11 H-pyrano[2,3-*f*]pyrido[3,2,1-ij]quinolin-11-one (NON), 2-(heptafluoropropyl)– 3-phenylquinoxaline (HEP), and 2,2,3,3,4,4,5,5,5-nonafluoro-N-(4-nitrophenyl)pentanamide (NNN) elicited significant antiandrogenic effects at relatively low concentrations. We further investigated the mechanism of AR inhibition and found that all three PFASs inhibited AR transactivation induced by testosterone through a competitive binding mechanism. We then examined the antiandrogenic effects of these PFASs on AR expression and its responsive genes. Consistently, these PFASs significantly decreased the expression of *PSA* and *FKBP5* and increased the expression of *AR*, similar to the effects elicited by a known competitive AR inhibitor, hydroxyflutamide. This suggests they are competitive antagonists of AR activity and western blot analysis revealed these PFASs decreased intracellular AR protein in androgen sensitive human prostate cancer cells. Hence, the findings presented here corroborate our published *in silico* approach and indicate these emerging PFASs may adversely affect the human endocrine system.

## Graphical Abstract

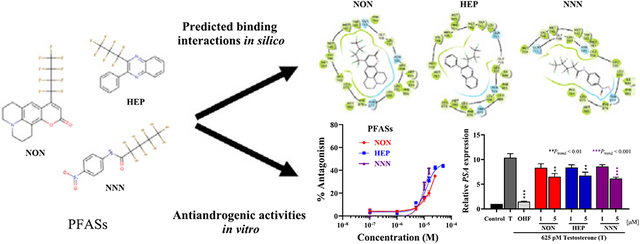

## 1. Introduction

Per- and poly-fluoroalkyl substances (PFASs) are a structurally diverse group of thousands of synthetic chemicals composed of fluorinated carbon chains. PFASs exhibit useful properties including high thermal and corrosion resistance, low friction performance, and stain repellency. As a result, PFASs are used globally in numerous industrial applications (flame retardants, surfactants, and textile coatings) and consumer products (furniture, food packaging, and non-stick cookware) (Buck et al., 2011; Herzke et al., 2012; D’eon and Mabury, 2011). In addition to being pervasive, the very stable chemical bond between the carbon and fluorine atoms makes them extremely persistent in the environment (Toskos et al., 2019) and bioaccumulative in humans and wildlife (Wang et al., 2017). For example, the most commonly studied long chain legacy PFASs, perfluorooctanesulfonic acid (PFOS) and perfluorooctanoic acid (PFOA), have been detected in many environmental metrices such as soil, surface water, and groundwater (Murakami et al., 2009). Apart from the environmental residues, PFASs have been found in drinking water and indoor dust (Harrad et al., 2019) at relatively high levels, which highlight the potential direct human exposure to these chemicals (Xu et al., 2020). The U.S. Environmental Protection Agency (U.S. EPA) reported the maximum levels of PFOS and PFOA in drinking water were at 7000 and 349 ng/L, respectively (Thomaidi et al., 2020). In addition, PFOS and PFOA have been found in indoor dust samples up to 140 and 83 ng/g, respectively (Harrad et al., 2019). Strikingly, PFOA and PFOS have also been found to be ubiquitous in human samples such as cord blood (Apelberg et al., 2007), plasma (Fromme et al., 2010), liver tissues (Olsen et al., 2003), and breast milk (Abdallah et al., 2020), indicating that the issue goes beyond an environmental concern. In an epidemiological study of mother-infant cohort, plasma levels of PFASs were detected at higher levels in 6- and 19-months old infants compared to mothers due to being breastfed, hand-to-mouth behavior and crawling on the ground (Fromme et al., 2010), suggesting infants are significantly exposed to PFAS toxicity. Given the dynamics of developmental processes during pregnancy, infancy, and childhood, exposure to PFASs during these periods is speculated to have the most pronounced negative health effects.

Adverse health effects resulting from PFAS exposure are a major public health concern. Studies have shown that exposure to PFASs has been linked to prostate cancer (Imir et al., 2021; Lundin et al., 2009), breast cancer (Tsai et al., 2020), liver disease (Bassler et al., 2019), and immunotoxicity (Pennings et al., 2016). Furthermore, several epidemiological studies have shown that exposure to legacy PFASs, namely PFOS and PFOA, is associated with endocrine disruption (Wang et al., 2019) including effects such as lower sperm quality (Joensen et al., 2009). Importantly, the androgen receptor (AR) has been implicated in several of these diseases and adversities (Gild et al., 2018; Basaria, 2014; O’Hara and Smith, 2015). For example, altered activation of AR is well known to contribute to lower sperm quality, spermatogenesis, infertility and prostate development (O’Hara and Smith, 2015). In addition, several epidemiological studies have shown that occupational PFAS exposure or living in PFAS contaminated areas (Hardell et al., 2014; Barry et al., 2013; Eriksen et al., 2009) is associated with increased risk of prostate cancer, suggesting the potential role of PFASs in prostate carcinogenesis.

Despite plans to restrict and eliminate long chain legacy PFASs including PFOA and PFOS (Wang et al., 2013, 2015; Lindstrom et al., 2011), thousands of PFASs exist but exposure and hazard for these remain largely unknown. Therefore, early identification of those that may interfere with bioactive molecules known to cause adverse outcomes associated with PFASs, such as the AR, is urgently needed. We speculate that there are uncharacterized PFASs that have the potential to disrupt human endocrine function. Indeed, in a previous *in silico* study, 5206 PFASs were screened from the EPA’s CompTox Chemicals Dashboard (a web-based database of curated compounds linked to chemical structures) against different binding sites on human AR (hAR) (Singam et al., 2020). The combination of docking-based screening and machine learning models identified 23 PFASs with strong predicted binding affinity against hAR (Singam et al., 2020).

In the present study, we sought to validate the AR biological activity of five commercially available PFASs namely (9-(nonafluorobutyl)– 2,3,6,7-tetrahydro-1 H,5 H,11 H-pyrano[2,3-*f*]pyrido[3,2,1-ij]quinolin-11-one (NON), 2-(heptafluoropropyl)– 3-phenylquinoxaline (HEP), 3-fluoro-4-{(*E*)-[4’-(heptafluoropropyl) [1,1’-biphenyl]– 4-yl]diazenyl} phenol (FLU), octafluoronaphthalene (OCT) and 2,2,3,3,4,4,5,5,5-nonafluoro-N-(4-nitrophenyl)pentanamide (NNN). These manufactured PFASs are categorized into the fluorinated aromatic substance subclass of PFASs (The Organisation for Economic Co-operation and Development OECD, 2018; Kwiatkowski et al., 2020). Specifically, NON, HEP, FLU, and NNN are classified in the PFAS subclass of ‘non-fluorinated aromatic rings with a fluorinated aliphatic side chain’, while OCT is classified as a PFAS in the subclass ‘fluorinated aromatic substance without a side chain’. We assessed androgenic and antiandrogenic activities of these chemicals *in vitro* using hAR mediated luciferase reporter gene assay. Of the five candidate AR ligands, three PFASs: NON, HEP, and NNN significantly disrupted the AR transactivation induced by testosterone. Furthermore, we investigated the mechanism of AR inhibition and the antiandrogenic effects of these PFASs on the expression of androgen responsive genes and intracellular AR protein levels in androgen sensitive human prostate cancer cells. Collectively, our findings increase awareness of potential endocrine disrupting outcomes caused by these emerging PFASs.

## 2. Materials and methods

### 2.1. Chemicals and reagents

All PFASs were dissolved into dimethyl sulfoxide (DMSO, ≥99.7% purity) from Fisher Scientific (Waltham, MA) (Table S1). Mifepristone (RU486, ≥98% purity), hydroxyflutamide (OHF, ≥98% purity), and testosterone (≥98% purity) were purchased from Sigma-Aldrich (St. Louis, MO). Enzalutamide (>98% purity) was obtained from Cayman Chemical (Ann Arbor, MI). 3-(4,5-dimethylthiazol-2-yl)– 2,5-diphenyltetrazolium bromide (MTT, ≥98% purity) was purchased from Amresco (Fountain Parkway Solon, OH).

### 2.2. Cell culture

The triple negative human breast cancer cell line (MDA-kb2) which highly expresses both endogenous AR and glucocorticoid receptor (GR), stably transfected with the murine mammalian tumor virus (MMTV) luciferase reporter gene construct, was obtained from the American Type Culture Collection (ATCC, Manassas, VA). Cells were maintained in Leibovitz’s medium (L-15, Gibco, Grand Island, NY) supplemented with 10% fetal bovine serum (FBS, Gemini, Bedford, MA) at 37 °C in a humidified incubator without CO_2_. The human prostate cancer cell line (VCaP) which expresses high levels of wild-type AR, was purchased from ATCC. Cells were cultured in phenol red-free Dulbecco’s Modified Eagle Medium (DMEM, Gibco, Grand Island, NY) supplemented with 10% FBS (Corning, Bedford, MA) and 1% L-glutamine (Gibco, Grand Island, NY) at 37 °C in a humidified incubator with 5% CO_2_. All PFASs had no good leaving groups such as carboxylic group (Went, 1988) and were dissolved in DMSO due to the high stability, good solubility and low cytotoxicity compared to those anticipated stabilities, solubilities and cytotoxicities in ethanol, dimethylformamide, or acetone at a concentration of 0.1% v/v (Jamalzadeh et al., 2016).

### 2.3. Cell viability assay

The cytotoxic effect of PFASs was measured by a colorimetric MTT assay as described previously (Tachachartvanich et al., 2020). Briefly, MDA-kb2 cells were plated at a density of 3.0 × 10^4^ cells/well in clear 96-well plates (Thermo Scientific, Waltham, MA). After 24 h incubation, cells were treated with PFASs at concentrations ranging from 0.1 to 100 μM in triplicate per treatment. The optical density was assessed at 570 nm with reference wavelength of 650 nm using microplate spectrophotometer (Tecan Infinite® 200PRO, Austria). All cytotoxicity experiments were performed side-by-side with the corresponding cell culture assays and repeated at least three times in a separate independent setup.

### 2.4. AR-mediated luciferase reporter gene assay

The MDA-kb2 cells were maintained prior to the luciferase reporter gene assay as described in the previous study (Tachachartvanich et al., 2020). Briefly, cells were maintained in L-15 hormone deprived media supplemented with 10% charcoal-dextran (CD) stripped FBS (Hyclone, USA) for 7 days prior to the AR-mediated luciferase reporter gene assay. Cells were plated at a density of 3.0 × 10^4^ cells/well in white 96-well plates (Thermo Scientific, Waltham, MA) and incubated for 24 h. Cell culture media containing a potent GR inhibitor (RU486) at 100 nM were used in all luciferase reporter gene assays to completely inhibit transactivation induced by glucocorticoids given AR and GR have the same homologous DNA-binding domains and can activate the MMTV promoter (Kolšek et al., 2015). In all assays, cells were treated with PFASs at concentrations ranging from 0.1 to 100 μM. For the assay used to assess potential androgenic effects, cells were treated with each test chemical for 24 h without the addition of testosterone. For the assay used to assess potential anti-androgenic effects, cells were co-cultured with each PFAS and 625 pM (~EC_50_) testosterone.

To further examine the mechanism of AR inhibition, cells were co-cultured with the highest non-cytotoxic concentration of each PFAS and different concentrations of testosterone ranging from 9.76 pM to 10 nM. After 24 h incubation, luciferase activity was assessed by a microplate luminometer (Tecan Infinite® 200PRO, Austria). All experiments were performed in triplicate wells and repeated at least three times in a separate independent setup. The minimum detection limit for luciferase was 19.5 pM testosterone.

### 2.5. RNA isolation and gene expression assay

VCaP cells were cultured in phenol red-free DMEM supplemented with 10% charcoal-dextran stripped FBS (10% CD FBS, Hyclone, USA) and 1% L-glutamine (Gibco, Grand Island, NY) for 3 days prior to PFAS treatment. Cells were plated at a density of 2.0 × 10^6^ cells/well in 6-well plates (CellStar, Monroe, NC) and incubated 24 h. Cells were then co-cultured with the highest noncytotoxic concentrations of PFASs and 625 pM testosterone for 24 h. After the incubation, total RNA was extracted using the RNeasy Mini Kit (Qiagen, Germantown, MD). Briefly, 2 μg of total RNA were reverse transcribed to cDNA using the SuperScript IV VILO (Thermo Scientific, Waltham, MA) and subjected to real-time PCR (Stratagene MxP3005, Agilent Technologies, Palo Alto, CA). The cDNA was amplified in 10 μL of SYBR Green real-time PCR Master Mixes (Thermofisher Scientific, Waltham, MA) according to the manufacturer’s protocol. Relative mRNA expression levels of androgen sensitive genes were evaluated using the following primers: *PSA* forward (5’-TCTGCGGCGGTGTTCTG-3’) and reverse (5’-GCCGACCCAGCAAGATCA-3’); *FKBP5* forward (5’-CGGAAAGGAGAGGGATATTCA-3’) and reverse (5’-CCACATCTCTGCAGTCAAACA-3’); and AR forward (5’-CAGTGGATGGGCTGAAAAAT-3’) and reverse (5’-GGAGCTTGGTGAGCTGGTAG-3’), and were normalized to the housekeeping gene glyceraldehyde-3-phosphate dehydrogenase (using *GAPDH* forward (5’-GGATTTGGTCGTATTGGG-3’) and reverse (5’-GGAAGATGGTGATGGGATT-3’ primers)). All primers were obtained from Integrated DNA Technologies (Coralville, IA). The fold change of the target genes was compared to the vehicle control using the 2^−ΔΔCt^ method (Livak and Schmittgen, 2001).

### 2.6. Protein extraction and immunoblotting analysis

The levels of intracellular AR protein were investigated in two different conditions: hormone-deprived (10% CD FBS) and normal (10% FBS) conditions. In the hormone deprived conditions, VCaP cells were cultured and treated as described above in the gene expression assay. To assess the effects of PFASs on the AR protein levels under normal conditions (10% FBS), cells were cultured in phenol red-free DMEM supplemented with 10% FBS (Corning, Bedford, MA) and 1% L-glutamine (Gibco, Grand Island, NY). Cells were plated at a density of 2.0 × 10^6^ cells/well in 6-well plates (CellStar, Monroe, NC) and incubated 24 h. Then, cells were treated with indicated concentrations of PFASs and enzalutamide (positive control) for 48 hr. After incubation, cells were gently washed with ice cold phosphate buffer saline and lysed in RIPA lysis buffer (Thermofisher Scientific, Waltham, MA) containing 1 × protease inhibitor (Thermofisher Scientific, Waltham, MA). Cell lysates were vortexed rigorously and incubated on ice for 30 min followed by centrifugation at 14,000g for 15 min at 4 °C. The protein concentration was determined by the BCA protein assay (Thermofisher Scientific, Waltham, MA). The protein lysates (30 μg) were incubated for 10 min at 95 °C with loading dye containing 2% of *β*-mercaptoethanol. Proteins were separated on the basis of size in sodium dodecyl sulfate (SDS) polyacrylamide gel electrophoresis followed by electro-transfer onto nitrocellulose membranes (0.2 μM, BIO-RAD, USA). The membrane was probed with the AR monoclonal antibody (MA5–13426 Thermo Scientific, MA, USA) and the secondary antibody goat anti-mouse IgG-horseradish peroxidase (HRP, Thermo Scientific, MA). Alpha tubulin monoclonal antibody conjugated with HRP (1E4C11, Thermo Scientific, MA) was used as a loading control. SuperSignal West Femto Maximum Sensitivity Substrate reagents (Thermo Scientific, MA) were added 2 min prior to the chemiluminescent blot visualization with the ChemiDoc Imaging System (BIO-RAD, USA). The intensity of protein bands was quantified with ImageJ software (US National Institutes of Health).

### 2.7. Statistical analysis

Data are expressed as means ± SEM of at least three experiments performed independently. Statistical comparisons between treatments and control were performed by one-way analysis of variance (ANOVA) followed by Dunnett’s multiple-comparison analysis and linear regression analysis to assess linear trends at a statistical threshold of p < 0.05 (GraphPad Prism version 8.4.0, GraphPad Software Inc., San Diego, CA, USA).

## 3. Results and discussion

### 3.1. Androgenic and antiandrogenic effects of PFASs

**Fig. 1.**
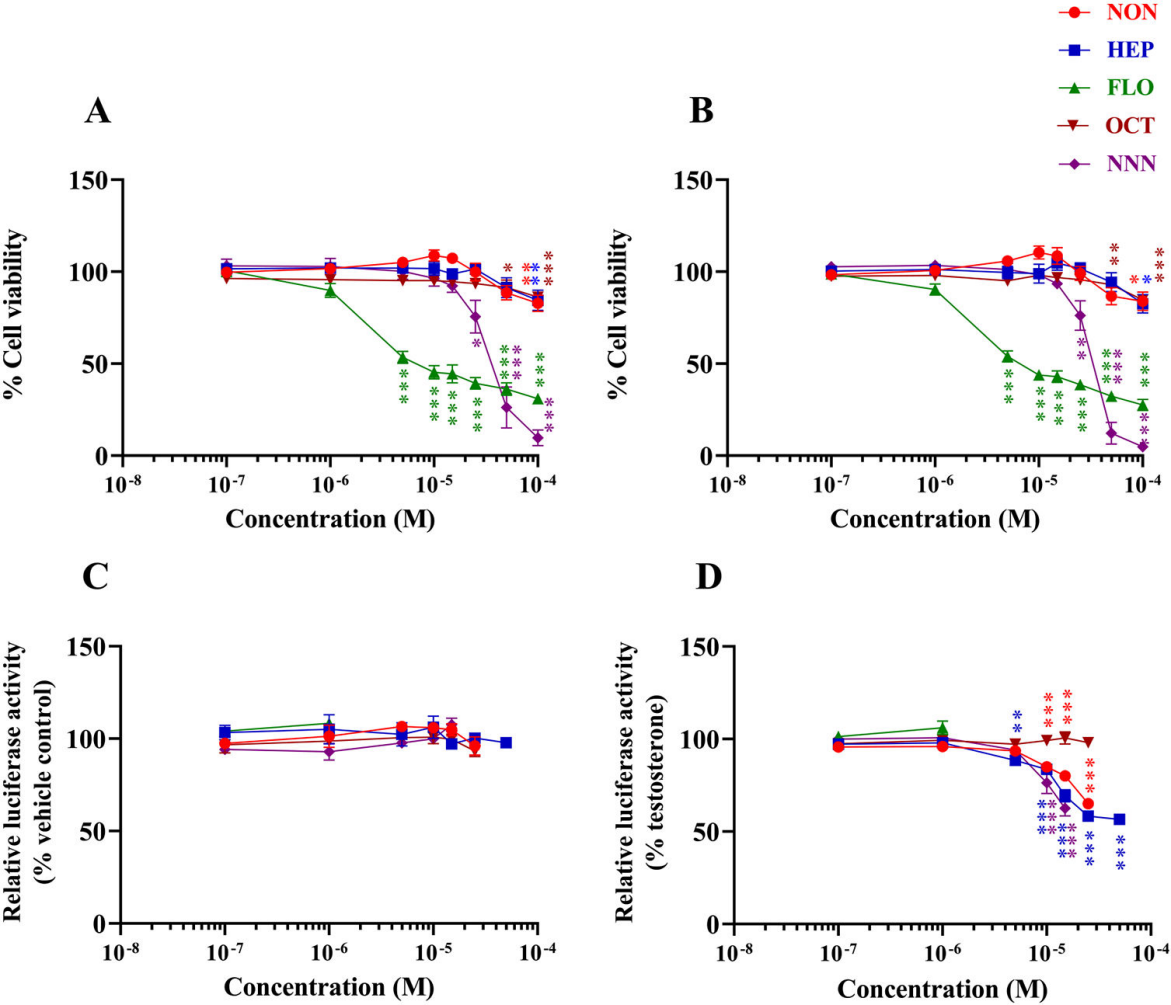
Androgenic and antiandrogenic effects of PFASs in the AR mediated luciferase reporter gene assay. MDA-kb2 cells were treated with various concentrations of PFASs ranging from 0.01 to 100 μM for 24 h. Cytotoxic effect of the PFASs in both the (A) androgenic and (B) antiandrogenic experiments was assessed by a colorimetric MTT assay. Concentrations of PFASs that caused a statistically significant reduction in cell viability were excluded from the analysis in the luciferase reporter gene assays that assessed the (C) androgenic and (D) antiandrogenic activities of PFASs. Values are expressed as the mean percentage of control ± S.E.M. from four independent experiments. Statistical analysis was performed using one-way ANOVA followed by a multiple comparison analysis with Dunnett’s test. Significance levels are represented with asterisk as following: *p < 0.05, **p < 0.01, ***p < 0.001 and ****p < 0.0001.

In the present study, we aimed to validate our previous *in silico* analysis which identified 23 emerging PFASs as potential AR ligands (Singam et al., 2020) by assessing androgenic and antiandrogenic activities with hAR mediated luciferase reporter gene assays. Of the 23 candidate PFASs identified, due to the lack of commercial availability, we were able to locate and obtain five commercially available PFASs: NON, HEP, FLO, OCT, and NNN (Table S1). To assure any detected ligand activity was not confounded by cytotoxicity, concentrations of PFASs that produced a statistically significant reduction in cell viability were excluded from the analysis (Fig. 1A-1B). None of the five PFASs caused a significant androgenic effect when tested alone (Fig. 1C). However, three PFASs inhibited the AR transactivation in a concentration-dependent manner (Fig. 1D). Even though NNN had the highest docking score (the lowest predicted binding affinity against AR among the test PFASs), it was a slightly more potent inhibitor of AR, followed by HEP and NON, with 20% relative inhibitory concentrations (RIC20) of 2.8, 3.1, and 10.5 μM, respectively (Table 1). These results indicate a prominent antiandrogenic effect posed by these PFASs at relatively low concentrations (Fig. 1D). For FLO, its relatively high cytotoxicity (Behr et al., 2018) may have masked any observable effect on luciferase transactivation. In addition, OCT is distinct from the other four PFASs in that it is a quite small planar aromatic hydrophobic molecule, and may have low AR specificity.

Even though toxicological data and endocrine disrupting potential of PFOA and PFOS are well established, data for emerging PFAS replacements is scarce. In an epidemiological study conducted in Denmark, men with high circulating levels of legacy PFASs such as PFOS and PFOA had a significantly lower number of normal spermatozoa (6.2 million) compared to men with lower PFOS and PFOA levels (15.5 million). This striking difference in the number of normal spermatozoa can be explained by the antiandrogenic effects posed by PFOS and PFOA (Joensen et al., 2009; Sifakis et al., 2017). It is important to note that there are inconsistent reports on the potential AR antagonism of PFOA. While some *in vitro* studies reported no antagonistic effect of PFOA on AR (Rosenmai et al., 2016), others found a significant antiandrogenic effect (Kjeldsen and Bonefeld-Jørgensen, 2013). For example, in an *in vitro* study, legacy PFASs such as PFOA, perfluorononanoate (PFNA), perfluorodecanoate (PFDA) and perfluorohexane sulfonate (PFHxS) exerted an antiandrogenic effect with RIC_20_ values around 10, 44, 24, and 19 μM, respectively (Kjeldsen and Bonefeld-Jørgensen, 2013). Alarmingly, when comparing the potency of antiandrogenic activities among PFASs, the RIC_20_ values of legacy PFASs are higher than those of the emerging PFASs tested herein. This provides further evidence that some emerging PFASs are not as safe as previously thought and may have even greater potential to cause serious endocrine disrupting hazards than their legacy predecessors. In addition, it is important to note that apart from the direct inhibition at the hormone receptor, endocrine disruptors can elicit adverse health effects through different mechanisms such as disrupted steroidogenesis (Rosenmai et al., 2016; La Merrill et al., 2020; Tachachartvanich et al., 2018). Further studies on the potential adverse effect of the emerging PFASs on steroidogenesis are warranted for more comprehensive hazard characterization.

### 3.2. PFASs inhibit AR through an apparent competitive binding mechanism

**Fig. 2.**
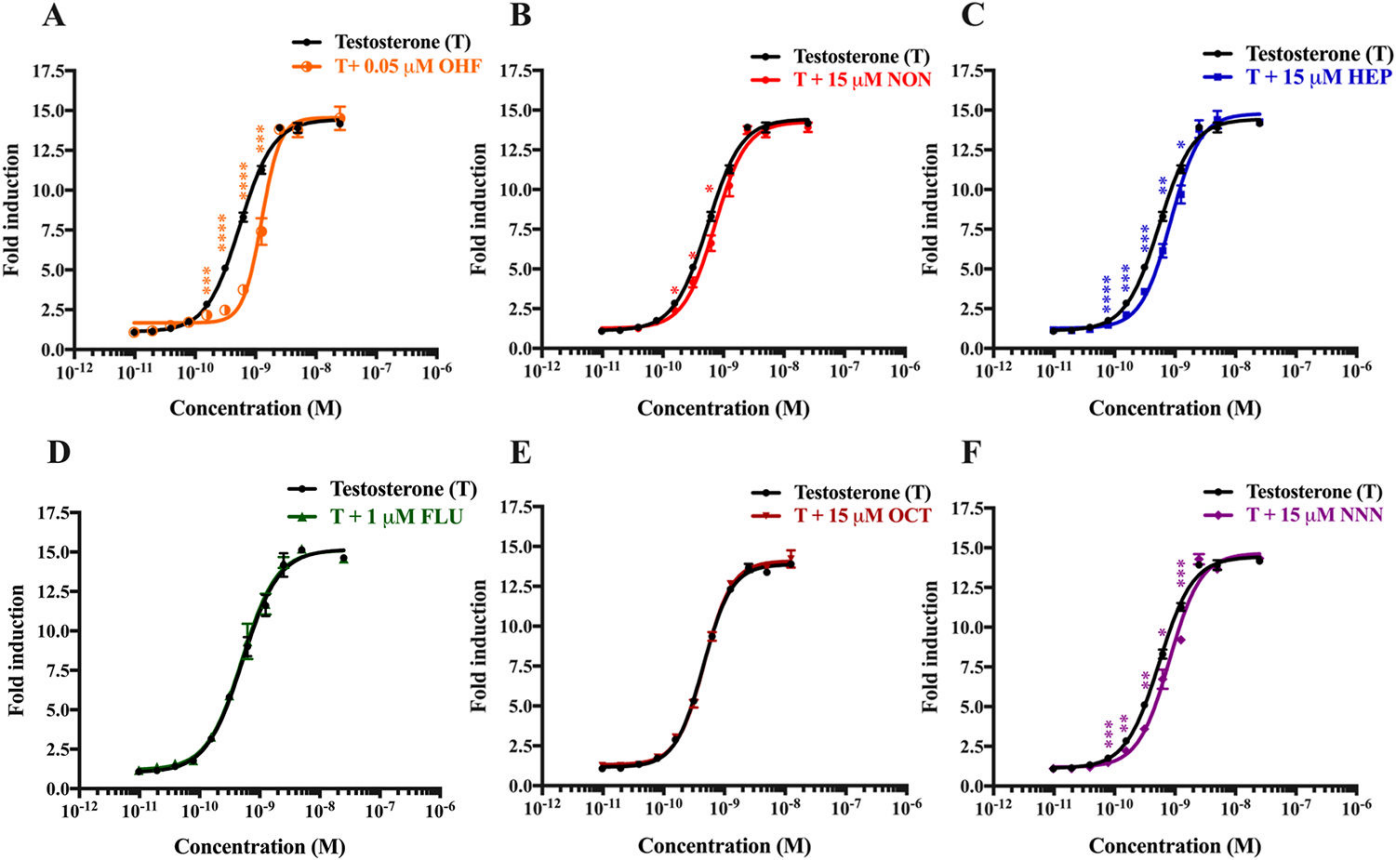
Competitive binding mechanism of PFASs against AR. MDA-kb2 cells were treated for 24 h with different concentrations of testosterone ranging from 9.7 pM to 10 nM (EC_50_ = ~550 pM) in the absence (black line) or presence (color line) of PFASs. Dose-response curves of testosterone in the presence of (A) 0.05 μM OHF (positive control), a well characterized competitive AR antagonist, (B) 15 μM NON, (C) 15 μM HEP, (D) 1 μM FLO, (E) 15 μM OCT, and (F) 15 μM NNN. The fold induction was compared to vehicle control (0.1% v/v DMSO). Values are expressed as the mean fold induction ± S.E.M. of three separate independent experiments. Statistical analysis was performed using Student’s t-test. Significance levels are represented with asterisk as following: *p < 0.05, **p < 0.01, ***p < 0.001 and ****p < 0.0001.

The two main mechanisms of hormone receptor inhibition elicited by endocrine disruptors are competitive and noncompetitive inhibition. In competitive inhibition, the receptor is inhibited when antagonists competitively bind to the ligand binding pocket (the same binding location as agonists) and inhibit the intrinsic activity of the receptor. In bioassays, competitive inhibitors typically generate a parallel shift of agonist dose response curves with no prominent effects on the maximal response (Vauquelin et al., 2002; Teng et al., 2013). In contrast, noncompetitive inhibition occurs when the antagonists bind the receptor at an allosteric site. In bioassays, noncompetitive inhibitors only suppress the maximal response but typically do not produce a shift of agonist dose-response curves (Tachachartvanich et al., 2020; Neubig et al., 2003). We speculated the emerging PFASs tested would inhibit AR through competitive inhibition as they were predicted computationally to bind the ligand binding domain of AR. To address this hypothesis, we investigated the mechanism of AR inhibition in a functional luciferase reporter gene assay. The dose response curve of testosterone co-cultured with OHF, a known competitive AR antagonist, is depicted in Fig. 2A. OHF showed the most parallel shift of dose response curve of testosterone with a half maximal effective concentration (EC_50_) of 550 pM. As expected, NON, HEP, and NNN caused a rightward parallel shift of the dose response curves with EC_50_ values of 722, 810, and 849 pM, respectively (Fig. 2B, C, and F). These trends are similar to testosterone co-cultured with OHF, indicating that these emerging PFASs inhibit AR binding to testosterone through competitive inhibition, corroborating the *in silico* analysis. Conversely, FLO and OCT, did not change the dose response curves even at the highest noncytotoxic concentrations (Fig. 2D and E), consistent with the results observed in the luciferase reporter gene assay that these chemicals did not exert antiandrogenic effects at noncytotoxic concentrations. Comparing the mechanism of AR inhibition with other environmental endocrine disruptors, similar to the binding mechanism of the test PFASs, bisphenol A (BPA) and bisphenol AF (BPAF) were found to inhibit AR transactivation through a competitive binding mechanism (Teng et al., 2013); however the anti-androgenic potency of BPA and BPAF is higher than the PFASs studied here.

### 3.3. PFASs form notable chemical interactions at the AR

**Fig. 3.**
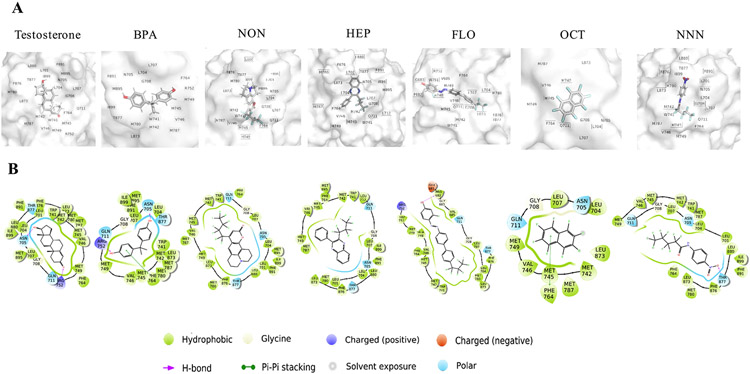
Docking poses of PFASs, testosterone (endogenous AR ligand), and BPA (antiandrogenic endocrine disruptor) formed the complex with the ligand binding domain of human AR (PDB: 3ZQT). (A) The location of the AR where the PFASs, testosterone, and BPA bind to is shown in the 3D structure. (B) Chemical interactions between the test chemicals and the key amino acid residues of AR are shown in the 2D structure. 2D and 3D structures were generated using Schrödinger and pymol packages, respectively.

Molecular docking is an efficient computational method used to examine intermolecular interactions at atomic levels between small molecules and nuclear hormone receptors (Zhang et al., 2015). The 2D and 3D structures of the five PFASs interacted with amino acids of hAR at the ligand binding domain are shown in Fig. 3. Moreover, NON, HEP, FLO, OCT, and NNN were predicted to bind at the ligand binding pocket with docking scores of −11.4, −10.74, −9.68, −9.22, and −8.3 kcal/mol, respectively (Table S1). This binding energy was facilitated by close proximity interactions of PFASs with numerous amino acid residues at the ligand binding domain of hAR, e.g. ALA748, ARG752, ASN705, GLN711, GLU681, GLY683, GLY708, LEU701, LEU704, LEU707, LEU873, LEU880, MET741, MET742, MET749, MET787, PHE764, PHE876, PRO682, TRP745, THR877, VAL685, and VAL746. Three of these amino acid residues in the ligand binding pocket of hAR namely, ARG752, ASN705 and THR877 play a pivotal role in stabilizing strong interactions between the receptor and endogenous androgens such as testosterone (Azhagiya Singam et al., 2019).

We next evaluated the nature of the interactions between the PFASs and amino acid residues. Three remarkable chemical bonds including hydrophobic, Pi-Pi stacking, and hydrogen bonding interactions were found to stabilize the binding between the PFASs and AR residues. For example, all five PFASs demonstrated a hydrophobic interaction with hydrophobic amino acids in close proximity. In addition, Pi-Pi stacking was observed in the complex between the ligand binding domain (PHE764) and one of the phenyl rings of OCT. Comparing OCT with other antiandrogens that have a similar binding mode to AR, *p*,*p*’-dichlorodiphenylethane (*p*,*p*′-DDE) has been identified as a weak anti-androgen (Freyberger and Ahr, 2004) and its binding mode with AR was predicted *in silico* in which pi-pi stacking was formed with the same residue (PHE764) as OCT (Azhagiya Singam et al., 2019). Among the intermolecular forces, the hydrogen bonding is considered a strong interaction, which was found in the hydroxyl group of FLO and the nitro group of NNN with PRO682 and THR877, respectively. It has been reported that environmental antiandrogens form hydrogen bond with AR at the key amino acid residues (ARG752, ASN705 and THR877) significantly inhibit AR interactions with testosterone. For example, BPA forms a hydrogen bond with the key amino acid residue and is a potent antiandrogen compared to other environmental antiandrogens that do not interact with these key amino acids (Conroy-Ben et al., 2018; Ermler et al., 2011). Interestingly, only NNN acts as a hydrogen bond acceptor with one of the key amino acids (THR877) at the ligand binding pocket, indicating this chemical can replace testosterone at the pocket site. This finding is in accordance with the results revealed in the luciferase and gene expression assays where NNN exerted the most potent anti-androgenic effects compared to other PFASs. Previous *in vitro* studies have reported that BPA suppressed AR transactivation by 50% at concentrations ranging from 3.8 to 10 μM (Conroy-Ben et al., 2018; Rosenmai et al., 2014); however, at 10 μM NNN inhibited AR transactivation around 25%, which suggests that BPA is more potent antiandrogenic than these PFASs.

### 3.4. PFASs notably alter the expression of androgen responsive genes

**Fig. 4.**
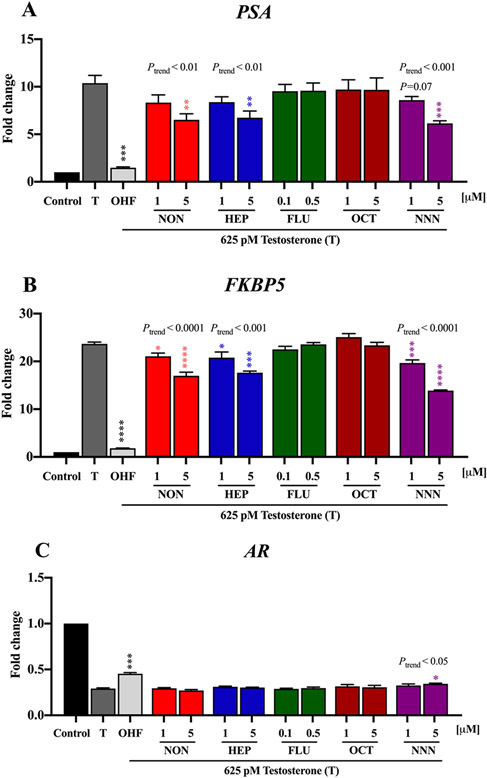
Antiandrogenic effect of PFASs on the expression of (A) *PSA*, (B) *FKBP5,* and (C) *AR* in VCaP human prostate cancer cells. Cells were cotreated with 625 pM testosterone and PFASs or 50 nM OHF (positive control) for 24 h under hormone deprived conditions (10% CD FBS). Values are expressed as the mean fold change ± S.E.M. from three independent experiments. Statistical analysis was performed using one-way ANOVA followed by a multiple comparison analysis with Dunnett’s test to compare the difference in fold change between exposed groups and the corresponding control (625 pM testosterone). Significance levels are represented with asterisk as following: *p < 0.05, **p < 0.01, ***p < 0.001, and ****p < 0.0001. The *P*_trend_ is determined based on linear regression analysis.

Upon binding of androgens, the AR functions as a ligand-dependent transcription factor that regulates the expression of androgen responsive genes (Ellison and Waller, 1978). We further investigated the endocrine disrupting effects of the emerging PFASs at the transcript level in highly sensitive AR responsive prostate cancer cells (VCaP). These cells highly express AR wild type and a wide array of known androgen-regulated genes such as *PSA*, *FKBP5*, and *AR*, which are biomarkers to evaluate functional and biological effects of antiandrogenic chemicals (Shaw et al., 2016; Yu et al., 2019). PSA and FKBP5 are transcriptionally upregulated by AR agonists whereas AR mRNA levels are inhibited by AR agonists via transcriptional and post-transcriptional mechanisms (Burnstein, 2005). Due to the high sensitivity of prostate cancer cells to antiandrogens, we selected lower concentrations of PFASs (1 and 5 μM) for further experiments in VCaP cell model. Consistent with the functional luciferase reporter gene assays, NON, HEP, and NNN significantly and concentration dependently decreased testosterone induced expression of *PSA* and *FKBP5*, which are known to play an important role in the dissolution of the seminal fluid coagulum (Balk et al., 2003) and modulate AR functions with heat shock proteins (Y. Li et al., 2011; L. Li et al., 2011), respectively (Fig. 4A and B). Among PFASs, only NNN significantly antagonized testosterone-induced downregulation of *AR* by inversely upregulating the expression of AR, similar to the effect exhibited by a known AR inhibitor, OHF (Fig. 4C). We compared the potency of antiandrogenic effect of PFASs with other environmental antiandrogens. Kharlyngdoh et al. (2015), reported that three brominated flame retardants (BFRs) allyl 2,4,6-tribromophenyl ether (ATE), 2-bromoallyl 2,4,6-tribromophenyl ether (BATE), and 2,3-dibromopropyl2,4,6-tribromophenyl ether (DPTE) significantly increased *AR* expression at 1 μM; however, NNN did not significantly affect *AR* expression at the same concentration. This suggests NNN is a weaker antiandrogen compared to BFRs. Likewise, all three BFRs significantly decreased testosterone induced *PSA* expression at 1 μM. However, the lowest observed effect concentration of PFASs on *PSA* expression is 5 μM, indicating that the antiandrogenic effect of the PFASs is lower than BFRs. Comparing magnitude of change in the androgenic gene expression after PFAS exposure, *FKBP5* and *PSA* genes are more sensitive to PFASs compared to *AR*. We highlight that *FKBP5* and *PSA* can serve as suitable markers for the assessment of antiandrogenic effects of PFASs in AR sensitive human prostate cancer cells.

### 3.5. The observed disruption of testosterone-induced androgen responsive gene expression by emerging PFASs is consistent with a direct inhibition of AR activity

**Fig. 5.**
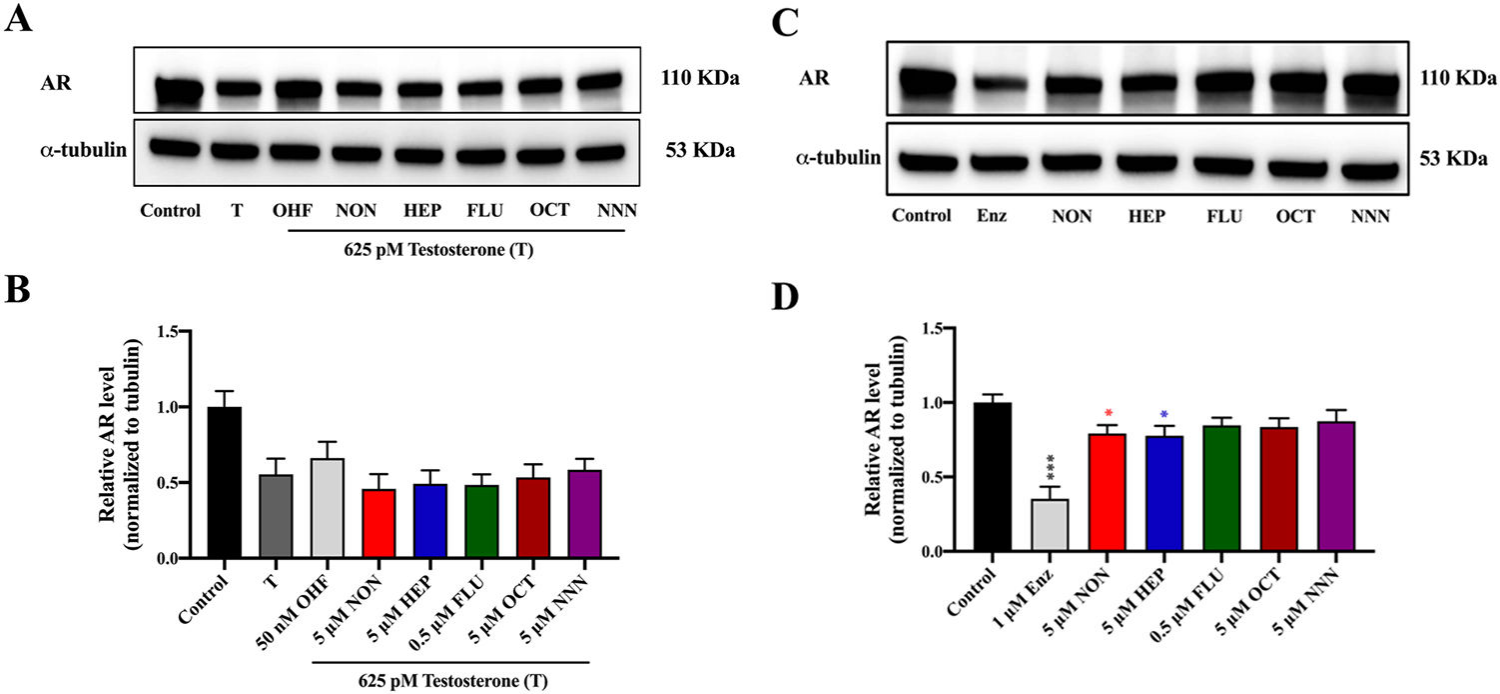
Antiandrogenic effect of PFASs on the AR protein levels in prostate cancer cells (VCaP). (A and B) cells were cotreated with 625 pM testosterone (T) and PFASs or 50 nM OHF (positive control) for 24 h under hormone deprived conditions (10% CD FBS). (C and D) cells were treated with PFASs or 1 μM enzalutamide (Enz, positive control) for 48 h under normal conditions (containing 10% intact, non-CD stripped FBS). AR proteins were detected using AR-specific antibody and α-tubulin was used as a loading control. Values are expressed as the mean fold change ± S.E.M. from three independent experiments. Statistical analysis was performed using one-way ANOVA followed by a multiple comparison analysis with Dunnett’s test to compare the difference in fold change between exposed groups and the corresponding control (625 pM testosterone for panel B) and (0.1% v/v DMSO for panel D). Significance levels are represented with asterisk as following: *p < 0.05 and * **p < 0.001.

Despite the antiandrogenic effects of NON, HEP, and NNN observed in both the luciferase and gene expression assays, concern has been identified regarding potential ligand-independent mechanisms as some environmental endocrine disruptors can reduce AR luciferase transactivation independent of ligand binding to AR. For example, pyrifluquinazon, a newly developed insecticide, exhibited antiandrogenic effects by promoting the degradation of cellular AR protein but did not directly inhibit the AR binding (Yasunaga et al., 2013). Based on our *in silico* prediction of binding affinity and luciferase AR competitive inhibition assay, we hypothesized that the test PFASs inhibited the AR via the ligand-dependent competitive inhibition mechanism. To demonstrate this, we further examined if the change in androgen responsive genes in the previous experiment was a consequence of the decline of AR protein. With identical cell culture conditions (hormone deprived condition: media containing 10% CD FBS) conducted in the gene expression assay, immunoblotting results revealed that none of the PFASs significantly changed the level of AR protein. This suggests the PFASs do not affect AR at the protein level and instead that the change in gene expression observed was mediated through the direct inhibition of PFASs on hAR (Fig. 5A and B).

### 3.6. PFASs significantly decrease intracellular AR protein levels under normal culture conditions (in media containing 10% intact, non-CD stripped FBS)

Research studies have shown that exposure to environmental or pharmaceutical antiandrogenic chemicals could reduce intracellular AR protein levels (Auvin et al., 2019; Cha et al., 2005; L. Li et al., 2011; Y. Li et al., 2011; Huang et al., 2019). For example, in VCaP cells, exposure to an antiandrogenic analogue of curcumin significantly decreased cellular AR protein in both time and concentration dependent manners (L. Li et al., 2011; Y. Li et al., 2011). Here, we examined if the five PFASs affected the cellular AR level in cells cultured in media containing 10% FBS. As expected, the positive control, enzalutamide, a well characterized pharmaceutical antiandrogen, significantly lowered cellular AR levels, consistent with previous reports (Pollock et al., 2016). Only NON and HEP significantly decreased the AR protein in the prostate cancer cells after 48 h exposure, consistent with NON and HEP as notable antiandrogenic PFASs, and prolonged exposure to these PFASs can significantly affect AR protein levels (Fig. 5C and D). However, NNN did not significantly affect cellular AR protein levels, indicating that the antiandrogenic effects exerted by NNN is mediated through a mechanism independent of effects on AR protein levels. While several studies have measured the levels of some of the short chain alternative PFASs such as F-53B, GenX, and FC-98 in the environment and humans (Li et al., 2020; Brandsma et al., 2019; Gebbink and van Leeuwen, 2020), numerous other emerging PFASs, including NON, HEP, and NNN, have no environmental fate information available. Such a knowledge gap has hindered the risk assessment for these compounds. Apart from the parent compounds, it is important to also assess the risk of the metabolites since the toxicity of the parent compounds may be different from their metabolites. Indeed, PFASs are comprised of a wide range of precursors that can be metabolized to possible toxic byproducts such as perfluroroalkyl acids (PFAA), which are structurally similar to their legacy predecessors. For example, metabolic hydrolysis of the amide bond of NNN can release PFAA: perfluoropentanoic acid and an aromatic amine, which have been associated with dermal toxicity (Han et al., 2020), liver damage, and genotoxicity (Bradshaw et al., 2018). This emphasizes the need to fully understand the toxicity of the parent compounds and their metabolites. In addition, exploring the use of these PFASs in other aspects such as starting reagents/intermediates for the synthesis of other PFASs derived compounds could provide suggestive information regarding the use of these emerging PFASs.

## 4. Conclusion

To the best of our knowledge, this is the first report assessing the biological activity of emerging PFASs against hAR, with evidence indicating that these emerging PFASs competitively inhibit the hAR from binding to testosterone in a concentration dependent fashion. Although, there are no studies to date that measure levels of these PFASs in the environmental matrices or humans, our findings imply that these emerging PFASs may affect the hazard of some androgen-related diseases given they competitively inhibit the hAR and alter the expression of androgenic genes at relatively low concentrations *in vitro*. More importantly, the potency of antiandrogenic effects of these emerging PFASs is relatively higher than their legacy predecessors reported in previous *in vitro* studies. Future research should investigate the residue levels of these newly identified antiandrogenic PFASs in humans and their associated health outcomes related to AR signaling pathway disruption such as infertility, cancer, and reproductive development.

## Supplementary Material

Supplementary Table 1

## Figures and Tables

**Table 1:** Androgenic and antiandrogenic effects of PFASs in the AR mediated luciferase reporter gene assay.

Chemicals	Activation		Inhibition	
	REC_20_ (M)	RLA^a^ (%)	REC_20_ (M)	RLA^b^ (%)
Testosterone	2.2 × 10^−10^	100	–	100
9-(Nonafluorobutyl)– 2,3,6,7-tetrahydro-1 H,5 H,11 H-pyrano [2,3-*f*]pyrido[3,2,1-ij]quinolin-11-one (NON)	NE	–	1.05 × 10^−5^	65.06
2-(Heptafluoropropyl)–3-phenylquinoxaline (HEP)	NE	–	3.08 × 10^−6^	56.61
3-Fluoro-4-((*E*)-[4’-(heptafluoropropyl) [1,1’-biphenyl]– 4-yl]diazinyl)phenol (FLO)	NE	–	NE	–
Octafluoronaphthalene (OCT)	NE	–	NE	–
2,2,3,3,4,4,5,5,5-Nonafluoro-N-(4 nitrophenyl)pentanamide (NNN)	NE	–	2.78 × 10^−6^	62.52

NE: no effect.

REC_20_: 20% relative effective concentration. The concentration of the test chemicals showing 20% of the agonistic activity of 1 × 10^−8^ M testosterone via AR.

RIC_20_: 20% relative inhibitory concentration. The concentration of the test chemicals showing 20% of the antagonistic activity of 6.25 × 10^−10^ M testosterone via AR.

RLA^a^: relative luciferase activity. Percentage of maximum activity of the test chemicals with 100% activity defined as the activity obtained from testosterone at 1 × 10^−8^ M.

RLA^b^: relative luciferase activity. Percentage of maximum inhibition of the test chemicals with 100% activity defined as the activity obtained from testosterone at 6.25 × 10^−10^ M.

## Novelty statement

- The significance and novelty of the work, with respect to existing literature
  This research is highly significant because an excess of 5000 PFAS chemicals have not undergone hazard assessment, yet PFASs are ubiquitous contaminants of drinking water throughout the world. The urgent need to identify hazardous PFASs is daunting, with the existing literature only evaluating the hazard potential of some tens of PFAS. We evaluated over 5200 PFASs to identify PFASs that can bind the human androgen receptor using novel molecular docking and machine learning techniques.
- Why the studied material should be considered "hazardous material".

PFASs were predicted to bind human androgen receptor, which is critical in reproduction and cancers. Three PFASs competitively inhibited human androgen receptor and decreased its function.

## HIGHLIGHTS

- PFASs exhibit antiandrogenic effects in vitro at relatively low concentrations.
- PFASs inhibit human androgen receptor through a competitive binding mechanism.
- PFASs disrupt the expression of androgen responsive genes in human prostate cancer cells.
- Prolonged exposure to PFASs decreases androgen receptor protein levels in human prostate cancer cells.
