# Supplementary material for: In Vitro characterization of the endocrine disrupting effects of per- and poly-fluoroalkyl substances (PFASs) on the human androgen receptor
Source: J Hazard Mater. Author manuscript; Available in PMC 2022 Nov 28. (PMC9705075; doi:10.1016/j.jhazmat.2022.128243)
Supplement: Supplementary Table 1 [file NIHMS1846557-supplement-Supplementary_Table_1.docx]

**Supplementary Information**

***In Vitro* Characterization of the Endocrine Disrupting Effects of Per- and Poly-fluoroalkyl Substances (PFASs) on the Human Androgen Receptor**

Phum Tachachartvanich^1,2^, Azhagiya Singam Ettayapuram Ramaprasad^3^, Kathleen A. Durkin^3^, J. David Furlow^4^, Martyn T. Smith^5^ and Michele A. La Merrill^1^

^1^Department of Environmental Toxicology, University of California, Davis 95616, California, USA.

^2^Laboratory of Environmental Toxicology, Chulabhorn Research Institute, Bangkok 10210, Thailand.

^3^Molecular Graphics and Computation Facility, College of Chemistry, University of California, Berkeley 94720, California, USA.

^4^Department of Neurobiology, Physiology and Behavior, University of California, Davis 95616, California, USA.

^5^Division of Environmental Health Sciences, School of Public Health, University of California, Berkeley 94720, California, USA.

***______________________________________________________________________________***

**Correspondence:* Michele A. La Merrill, Ph.D., Associate Professor, Department of Environmental Toxicology, University of California at Davis, CA 95616, USA. E-mail: mlamerrill@ucdavis.edu.

Number of pages: 2

Number of tables: 1

**1. Table**

**Table S1.** Binding energy (kcal/mol) of the commercially available PFASs in a complex with the human AR at the ligand binding pocket (LBP).

| **Name** | **Chemical structure** | **Supplier** | **Catalog #** | **CAS #** | **%Purity** | **Binding energy** | **Rank #** |
| --- | --- | --- | --- | --- | --- | --- | --- |
| Testosterone (endogenous AR ligand) | 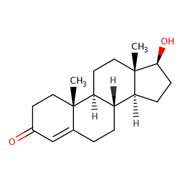  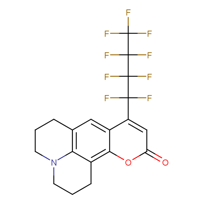 | Sigma | T-037 | 58-22-0 | >96% | -11.70 | N/A |
| 9-(Nonafluorobutyl)-2,3,6,7-tetrahydro-1H,5H,11H-pyrano[2,3-f]pyrido[3,2,1-ij]quinolin-11-one (NON) |  | Mcule, Inc. | 8407797723 | 355822-08-1 | >90% | -11.40 | 2 |
| 2-(Heptafluoropropyl)-3-phenylquinoxaline (HEP) | 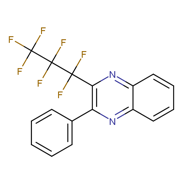  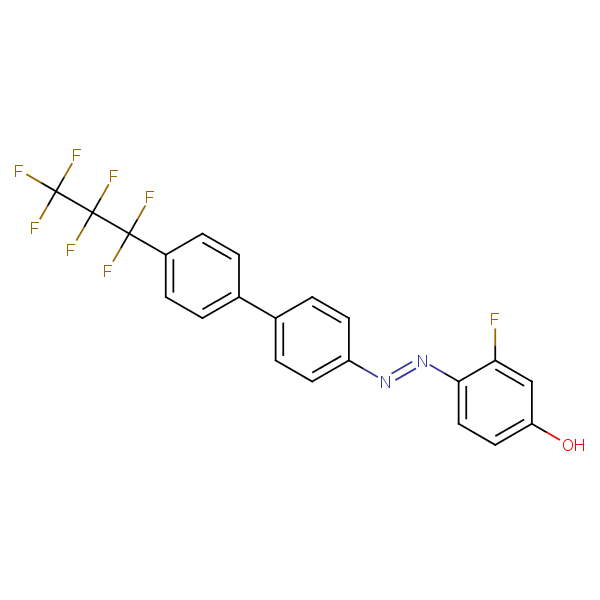 | TimTec LLC | ST099182 | 69492-70-2 | >90% | -10.74 | 5 |
| 3-Fluoro-4-{(E)-[4'-(heptafluoropropyl) [1,1'-biphenyl]-4-yl]diazenyl}phenol (FLU) |  | Mcule, Inc. | 6605399363 | 113448-90-1 | >90% | -9.68 | 13 |
| Octafluoronaphthalene (OCT) | 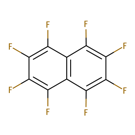  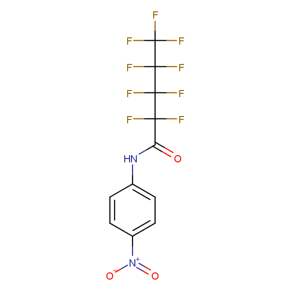 | Sigma | 248061 | 313-72-4 | >96% | -9.22 | 15 |
| 2,2,3,3,4,4,5,5,5-Nonafluoro-N-(4 nitrophenyl)pentanamide (NNN) |  | Mcule, Inc. | 8423892622 | 150333-62-3 | >90% | -8.30 | 22 |
